# 伴RUNX1单位点与多位点基因突变急性髓系白血病患者的临床特征及预后

**DOI:** 10.3760/cma.j.cn121090-20251020-00473

**Published:** 2026-06

**Authors:** 畅 李, 雅雪 吴, 骁 马, 苏宁 陈, 德沛 吴, 晓慧 胡

**Affiliations:** 1 苏州大学附属第一医院血液科，江苏省血液学研究所，国家血液系统疾病临床医学研究中心，苏州 215006 Department of Hematology, the First Affiliated Hospital of Soochow University, Jiangsu Institute of Hematology, National Clinical Research Center for Hematologic Diseases, Suzhou 215006, China; 2 苏州弘慈血液病医院血液科，苏州 215128 Soochow Hopes Hematology Hospital, Suzhou 215128, China

**Keywords:** 基因，RUNX1, 白血病，髓系，急性, 基因突变, 预后, Gene, RUNX1, Leukemia, Gene mutation, Prognosis

## Abstract

**目的:**

比较伴RUNX1单位点与多位点突变急性髓系白血病（AML）患者的临床特征及预后。

**方法:**

本研究为回顾性队列研究。选取2017年6月至2023年6月于苏州大学附属第一医院诊治的145例伴RUNX1突变AML患者。其中单位点突变患者123例，多位点突变患者22例，主要观察指标包括总生存（OS）时间、无复发生存（RFS）时间及首疗程诱导缓解率（完全缓解率和血细胞未完全恢复的完全缓解率之和）。

**结果:**

全部145例RUNX1突变阳性的AML患者中，男87例，女58例，中位年龄49（17～78）岁，单位点突变患者和多位点突变患者的年龄、性别、初诊时的血细胞计数、染色体核型、融合基因、预后分层等特征差异均无统计学意义（均*P*>0.05）。单位点突变患者共突变基因中位数为3（0～11）个，多位点突变患者共突变基因中位数为5（2～10）个，后者多于前者（*P*＝0.001），但两组间共突变基因谱分布差异无统计学意义（*P*>0.05）。两组首疗程诱导缓解率（53.3％对50.0％，*P*＝0.819）、中位OS时间（45.67个月对36.53个月，*P*＝0.942）及中位RFS时间（17.13个月对21.17个月，*P*＝0.679）差异均无统计学意义。单位点突变组接受异基因造血干细胞移植（allo-HSCT）患者的3年OS率及RFS率分别为74.7％和58.5％，显著高于未移植患者的31.2％和27.9％（均*P*<0.001）；多位点突变组接受allo-HSCT患者的3年OS率及RFS率分别为69.2％和69.2％，亦显著高于未移植患者的33.3％和0（*P*＝0.003及*P*＝0.001）。多因素Cox回归分析显示，allo-HSCT是两组患者OS（单位点突变组：*HR*＝0.256，*P*<0.001；多位点突变组：*HR*＝0.164，*P*＝0.043）及RFS（单位点突变组：*HR*＝0.528，*P*＝0.025；多位点突变组：*HR*＝0.142，*P*＝0.030）的独立保护因素。

**结论:**

伴RUNX1单位点突变和多位点突变的AML患者在临床特征、治疗反应及远期预后上无明显差异，allo-HSCT能有效改善两组患者的生存结局。

## Abstract (en)

**Objective:**

To compare the clinical characteristics and prognosis of patients with acute myeloid leukemia（AML）harboring single-site versus multi-site mutations in the RUNX1 gene.

**Methods:**

This study was a retrospective cohort study. 145 patients with RUNX1-mutated AML treated at the First Affiliated Hospital of Soochow University between June 2017 and June 2023 were selected. Single-site RUNX1 mutations were observed in 123 patients, whereas multi-site mutations were detected in 22 patients. The outcomes of interest included overall survival（OS）, recurrence-free survival（RFS）, and the first-course induction remission rate, defined as the sum of complete remission and complete remission with incomplete hematologic recovery.

**Results:**

A total of 145 patients with RUNX1-mutated AML were included, including 87 males and 58 females, with a median age of 49 years（range, 17–78 years）. The two groups showed no significant differences in age, sex composition, peripheral blood counts at diagnosis, karyotype, fusion genes, or risk stratification（all *P*>0.05）. The median number of co-occurring gene mutations was 3（range, 0–11）in the single-site group and 5（range, 2–10）in the multi-site group, indicating a significantly higher number in the latter（*P*＝0.001）. However, the distribution of co-occurring mutations did not differ significantly between groups（*P*>0.05）. There were no significant between-group differences in the first-course induction remission rate（53.3％ *vs* 50.0％, *P*＝0.819）, median OS（45.67 *vs* 36.53 months, *P*＝0.942）, or median RFS (17.13 *vs* 21.17 months, *P*＝0.679）. In the single-site mutation group, patients undergoing allogeneic hematopoietic stem cell transplantation（allo-HSCT）achieved 3-year OS and RFS rates of 74.7％ and 58.5％, respectively, significantly higher than the 31.2％ and 27.9％ among those without transplantation（both *P*<0.001）. In the multi-site mutation group, the 3-year OS and RFS rates were 69.2％ and 69.2％, respectively, among allo-HSCT recipients, significantly higher than 33.3% and 0, respectively, among nonrecipients（*P*＝0.003 and *P*＝0.001, respectively）. Multivariate Cox proportional hazards analysis identified allo-HSCT as an independent protective factor for OS（single-site group: *HR*＝0.256, *P*<0.001; multi-site group: *HR*＝0.164, *P*＝0.043）and RFS (single-site group: *HR*＝0.528, *P*＝0.025; multi-site group: *HR*＝0.142, *P*＝0.030）.

**Conclusion:**

Patients with AML harboring single-site and multi-site RUNX1 mutations did not differ significantly in clinical characteristics, treatment response, or long-term prognosis. Allo-HSCT was associated with improved survival outcomes in both groups.

急性髓系白血病（AML）是一种源于造血干细胞的异质性恶性克隆性疾病，以骨髓及外周血中原始细胞的克隆性增殖、正常骨髓造血功能受到抑制为特征。基因突变所致的蛋白质异常表达与造血细胞分化过程中的异常增殖和停滞相关。经典的“双打击模型”认为，第一类突变激活激酶信号通路，导致细胞过度增殖；第二类突变（如RUNX1）干扰转录因子功能，阻碍造血分化，二者协同促进白血病的发生。RUNX1基因可通过体细胞点突变或染色体易位导致功能失调，干扰造血干细胞的正常增殖及髓系分化，进而诱发血液系统恶性肿瘤[1]。随着二代测序技术在临床的开展，体细胞突变分析使AML的诊断和分型日益精准。RUNX1通常被认为是AML患者预后不良的分子标志[2]，2022年欧洲白血病网（ELN）指南已将RUNX1列为骨髓增生异常相关基因，归入预后不良组[3]。以往的研究多聚焦于RUNX1野生型与突变型的比较，本文旨在比较RUNX1单位点突变及多位点突变患者的临床特征及预后差异。

## 病例与方法

1. 病例资料：本研究为回顾性队列研究，选取2017年6月至2023年6月于苏州大学附属第一医院就诊的145例初诊伴RUNX1突变AML患者。所有患者均根据骨髓细胞形态学、免疫分型、细胞遗传学及分子生物学（MICM）进行诊断、分型和危险度分层，诊断标准参照《成人急性髓系白血病（非急性早幼粒细胞白血病）中国诊疗指南（2023年版）》[4]。本研究通过苏州大学附属第一医院医学伦理委员会批准［批件号：（2026）伦审批第842号］。

2. 基因突变的检测方法：提取初诊AML患者骨髓液3～5 ml，分离骨髓单个核细胞，抽提基因组DNA，采用探针捕获建库方法Nextseq550系统进行脱氧核糖核酸测序，平均测序深度1 000×以上，分析所有数据质量满足Q30，本检测方法平均突变的检出灵敏度为3％，设定等位基因变异频率（VAF）截断值为3％，检测基因为《二代测序技术在血液肿瘤中的应用中国专家共识（2018版）》推荐检测的基因[5]，将携带至少一个合格RUNX1突变的患者纳入分析，依据每例患者样本中检出的RUNX1基因突变位点个数，将患者分为单位点突变组（1个突变位点）和多位点突变组（≥2个突变位点）。

3. 治疗方案：诱导治疗采用标准剂量化疗［IA（去甲氧柔红霉素+阿糖胞苷）、DA（柔红霉素+阿糖胞苷）］、去甲基化药物（HMA，地西他滨/阿扎胞苷）单用或联合化疗（联合预激方案/低剂量化疗）、维奈克拉联合去甲基化药物（VEN-HMA）、预激方案CAG（阿克拉霉素+阿糖胞苷+G-CSF）。达完全缓解（CR）的患者后续接受中大剂量阿糖胞苷单药巩固治疗（3～4个疗程）或异基因造血干细胞移植（allo-HSCT）。未达CR的患者采用原方案或者更换其他方案行再次诱导治疗。单位点突变组60例、多位点突变组13例接受了allo-HSCT，移植前预处理主要采用改良Bu-Cy方案（白消安+环磷酰胺）[6]。

4. 疗效评价和随访：CR定义为骨髓原始细胞<5％，无外周血原始细胞，髓外病灶消失，中性粒细胞绝对计数（ANC）≥1.0×10^9^/L，PLT≥100×10^9^/L；完全缓解伴血细胞未完全恢复（CRi）定义为骨髓原始细胞<5％，外周血原始细胞及髓外病灶消失，ANC<1.0×10^9^/L，或PLT<100×10^9^/L；缓解率定义为CR率+CRi率。总生存（OS）时间定义为从确诊日期至死亡（任何原因）或末次随访日；无复发生存（RFS）时间定义为首次缓解至复发或者死亡（任何原因），无复发或死亡以末次随访日计算。患者随访采用电话、住院及门诊病历查询等方式，从患者确诊随访至2025年6月30日。

5. 统计学处理：采用SPSS 25.0软件进行统计分析，GraphPad Prism 10.0绘制图表。分类资料以频数和百分比表示，组间比较采用卡方检验或Fisher精确检验；连续性变量资料采用Shapiro-Wilk进行正态性检验，符合正态分布者以均数±标准差表示，组间比较采用*t*检验，不符合正态分布者以中位数（范围）表示，组间比较采用Mann-Whitney *U*非参数检验。生存分析采用Kaplan-Meier法绘制生存曲线，Log-rank检验比较组间生存率的差异。采用Cox回归模型进行单因素分析及多因素分析，多因素分析中对相关混杂因素进行校正，评估各变量对OS和RFS的独立影响，结果以风险比（*HR*）及95％置信区间（*CI*）表示，*P*<0.05为差异有统计学意义。稳健性分析：鉴于单位点突变组与多位点突变组样本量差异悬殊（123例对22例），对分类变量的组间比较，采用Bootstrap法（1 000次重抽样）估计比值比（*OR*）的95％ *CI*；对Cox回归分析，采用Bootstrap法（1 000次重抽样）估计风险比（*HR*）的95％*CI*，以验证结果的稳健性。

## 结果

1. 初诊时的临床特征：共纳入145例RUNX1突变的AML患者，其中男87例，女58例，中位年龄49（17～78）岁，初诊时中位WBC 6.6（0.3～170.3）×10^9^/L，HGB 76.0（20.0～181.0）g/L，PLT 45（5～680）×10⁹/L，骨髓原始细胞比例56.8％（4.5％～98.5％）。单位点突变患者123例（84.8％），多位点突变患者22例（15.2％）。两组患者在血常规、骨髓原始细胞比例、染色体核型及融合基因等方面差异均无统计学意义（表1）。

**表1:** 伴RUNX1单位点与多位点基因突变急性髓系白血病患者的临床特征比较

临床特征	单位点突变 （123例）	多位点突变 （22例）	*P*值
年龄［岁，*M*（范围）］	50（17～78）	46（29～77）	0.995
男性［例（％）］	75（61.0）	12（54.5）	0.639
WBC［×10^9^/L，*M*（范围）］	7.3（0.3～162.7）	5.3（0.8～170.3）	0.275
HGB［g/L，*M*（范围）］	74（20～181）	82.5（41～141）	0.448
PLT［×10^9^/L，*M*（范围）］	43（5～680）	56（7～156）	0.980
骨髓原始细胞［％，*M*（范围）］	56.8（4.5～98.5）	60.0（10.0～97.0）	0.768
共突变基因个数［*M*（范围）］	3（0～11）	5（2～10）	0.001
染色体核型［例（％）］			
inv（16）（p13q22）	2（1.6）	0（0）	1.000
–7	1（0.8）	0（0）	1.000
正常核型	74（60.2）	16（72.7）	0.342
复杂	8（6.5）	1（4.6）	1.000
5q–	1（0.8）	0（0）	1.000
t（8;21）（q22;q22）	2（1.6）	0（0）	1.000
t（9;22）（q34;q11）	1（0.8）	0（0）	1.000
单体核型	4（3.3）	0（0）	1.000
其他异常	30（24.4）	5（22.7）	1.000
融合基因［例（％）］			
阴性	111（90.3）	21（95.5）	0.692
RUNX1::RUNX1T1	4（3.3）	0（0）	1.000
BCR::ABL1	2（1.6）	0（0）	1.000
CBFB::MYH11	2（1.6）	0（0）	1.000
MLL-PTD	1（0.8）	0（0）	1.000
MLL::ELL	2（1.6）	0（0）	1.000
TLS::ERG	1（0.8）	0（0）	1.000
NUP98::HOXA9	0（0）	1（4.5）	0.152
预后危险度［例（％）］			
良好	14（11.4）	0（0）	0.129
中等	28（22.8）	7（31.8）	0.418
不良	81（65.9）	15（68.2）	1.000

2. RUNX1突变特征及其对预后的影响：单位点突变组中位VAF为42.6％（1.6％～93.4％），多位点突变组为30.8％（1.4％～51.3％），以VAF的中位数40.0％为分界，高VAF组（≥40.0％）中位OS时间为23.23（0.27～102.90）个月，3年OS率为45.4％，低VAF组（<40.0％）的中位OS时间为47.93（0.10～103.10）个月，3年OS率为61.7％，两组差异无统计学意义（*P*＝0.398）。高VAF组诱导缓解率为52.4％，低VAF组为52.7％，二者差异无统计学意义（*P*＝1.000）。总体患者在突变类型分布上，错义突变78例，移码突变53例，无义突变14例。单位点突变组中错义突变最为常见（52.0％），其次为移码突变（37.4％），无义突变比例最低（10.6％）；多位点组中错义突变比例更高（63.6％），其次为移码突变（31.8％），无义突变比例最低（4.5％），两组分布差异无统计学意义（*P*＝0.519）。生存分析显示，错义、移码、无义突变组的中位OS时间分别为39.0、39.2和15.2个月，3年OS率分别为52.1％、54.6％和50.0％，组间差异无统计学意义（*P*＝0.934）。三组缓解率分别为58.7％、49.1％和35.7％，差异亦无统计学意义（*P*＝0.244）。

3. RUNX1共突变基因特征：单位点突变组共突变基因中位数为3（0～11）个，多位点突变组共突变基因中位数为5（2～10）个，多位点突变组显著高于单位点突变组（*P*＝0.001）。单位点突变组最常见的共突变基因为FLT3（31.7％，39/123），其他依次为DNMT3A（23.6％，29/123）、NRAS（17.8％，22/123）、ASXL1（17.8％，22/123）、BCOR（15.4％，19/123）、WT1（13.8％，17/123）及IDH2（13.0％，16/123）。多位点突变组最常见的共突变基因为ASXL1（27.3％，6/22），其次为FLT3、WT1、NRAS（各22.7％，5/22）。两组间共突变基因分布差异无统计学意义（*P*>0.05，图1）。

**图1:**
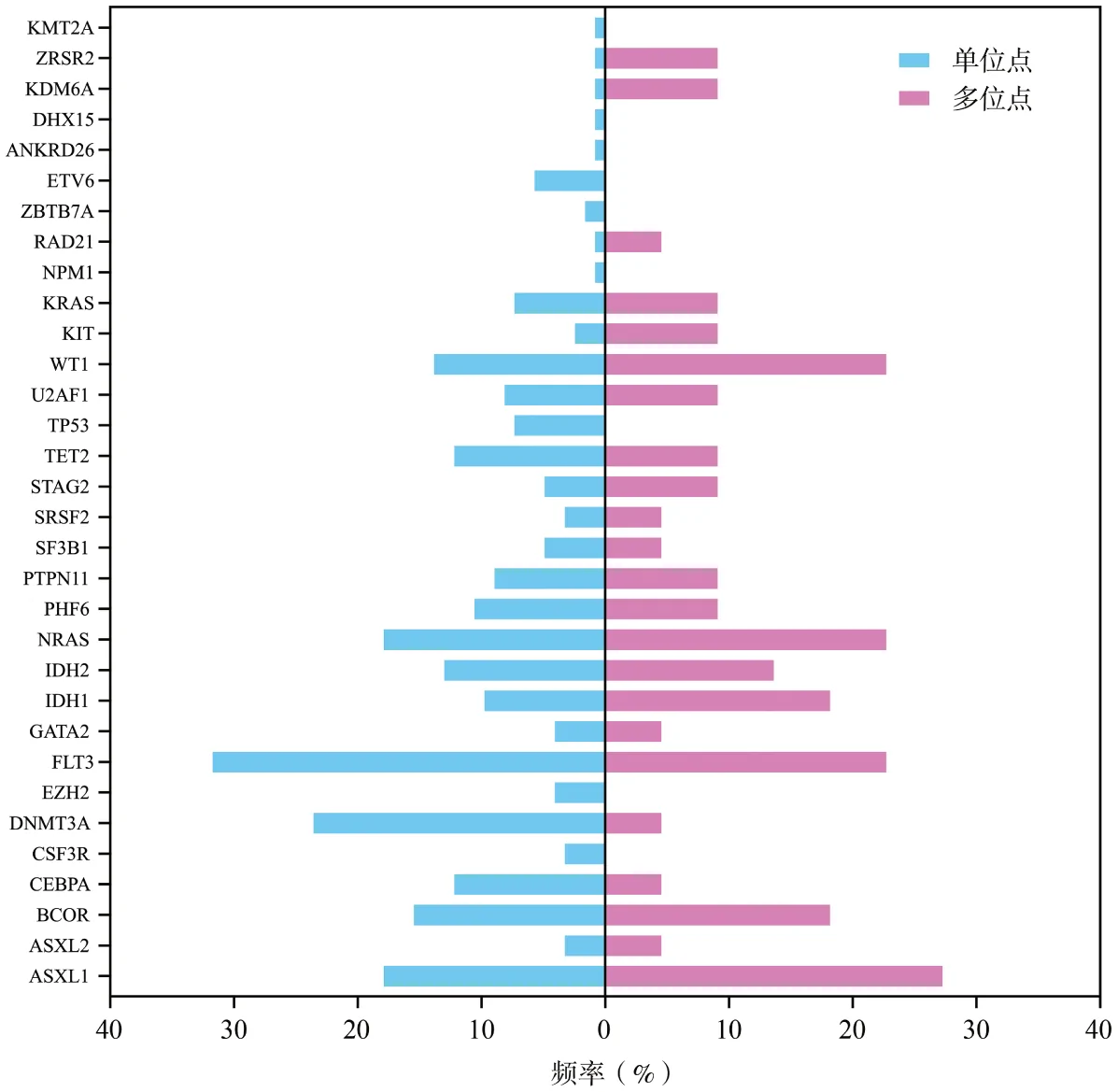
伴RUNX1单位点与多位点基因突变急性髓系白血病患者共突变基因分布

4. 诱导化疗反应：总体患者的首疗程诱导化疗的缓解率为52.8％，单位点突变组与多位点突变组分别为53.3％和50.0％，差异无统计学意义（*P*＝0.819）。Bootstrap结果显示，*OR*的95％ *CI*为0.430～3.188，包含1，提示该阴性结果稳健可靠。分层分析显示，单位点突变组与多位点突变组患者对不同诱导化疗方案的缓解率差异均无统计学意义（*P*＝0.741，*P*＝0.247），接受VEN-HMA治疗患者的缓解率（62.2％）相对较高（表2）。

**表2:** 不同治疗组患者疗效比较［例（％）］

治疗方案	总体		单位点突变组		多位点突变组	
	例数	CR+CRi	例数	CR+CRi	例数	CR+CRi
标准剂量化疗	47	23（48.9）	44	23（52.3）	3	0（0）
HMA联合化疗	44	24（54.5）	34	19（55.9）	10	5（50.0）
VEN-HMA	37	23（62.2）	30	18（60.0）	7	5（71.4）
去甲基化药物	8	3（37.5）	6	2（33.3）	2	1（50.0）
预激方案	5	2（40.0）	5	2（40.0）	0	0（0）

*P*值		0.606		0.741		0.247

**注** CR：完全缓解；CRi：血细胞未完全恢复的完全缓解；HMA：去甲基化药物；VEN：维奈克拉。4例单位点突变组患者治疗方案信息缺失

5. 生存分析：随访截至2025年6月30日，总体患者的中位OS时间为39.20（0.10～103.10）个月，3年OS率为51.6％。中位RFS时间为18.67（0～118.80）个月，3年RFS率为43.3％。单位点突变组与多位点突变组中位OS时间分别为45.67（0.10～103.10）个月和36.53（0.27～86.20）个月，3年OS率分别为51.5％和48.5％，差异无统计学意义（*P*＝0.942，图2A）。两组中位RFS时间分别为17.13（0～118.80）个月和21.17（0～79.80）个月，3年RFS率分别为43.1％和43.8％，差异亦无统计学意义（*P*＝0.679，图2B）。单因素Cox回归分析显示，以单位点组为参照，多位点突变未显著增加死亡或复发风险（OS: *HR*＝0.977，95％ *CI:* 0.528～1.810，*P*＝0.942；RFS: *HR*＝1.135，95％ *CI*: 0.616～2.090，*P*＝0.685），与Log-rank检验结果一致。稳健性分析：鉴于两组样本量悬殊（单位点组123例，多位点组仅22例），可能影响统计效力，Bootstrap法验证（1 000次重抽样）显示，OS的*HR* 95％ *CI*为0.53～1.97，RFS的*HR* 95％ *CI*为0.64～2.26，均包含1，且与常规分析一致，表明结论稳健可靠。

**图2:**
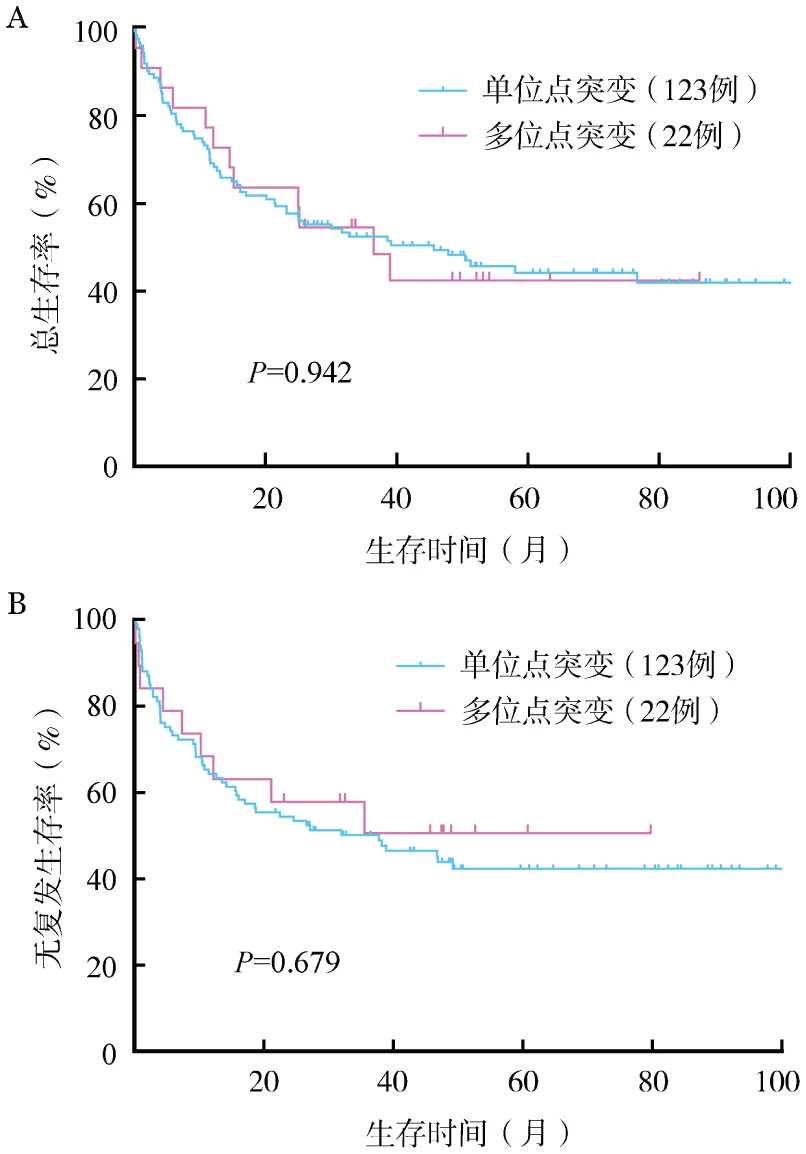
伴RUNX1单位点与多位点基因突变急性髓系白血病患者总生存（A）及无复发生存（B）曲线

6. allo-HSCT对两组患者生存率的影响：145例伴RUNX1突变AML患者中，73例接受allo-HSCT，其中单位点突变组60例（48.8％），多位点突变组13例（59.1％），移植时缓解率分别为88.3％和84.6％。单位点突变组接受移植患者的中位OS时间未达到，中位RFS时间为49.27（0～100.77）个月，3年OS率和RFS率分别为74.7％和58.5％；未移植患者中位OS时间为11.63（0.10～102.20）个月，中位RFS时间为9.47（0～118.80）个月，3年OS率和RFS率分别为31.2％和27.9％。两组OS和RFS差异有统计学意义（均*P*<0.001）（图3A、B）。多位点突变组移植患者的中位OS时间及中位RFS时间均未达到，3年OS率和RFS率分别为69.2％和69.2％；未移植患者的中位OS时间为14.60（0.27～39.00）个月，中位RFS时间为0.67（0～35.57）个月，3年OS率及RFS率分别为33.3％和0。两组OS、RFS差异有统计学意义（*P*＝0.003，*P*＝0.001）（图3C、D）。

**图3:**
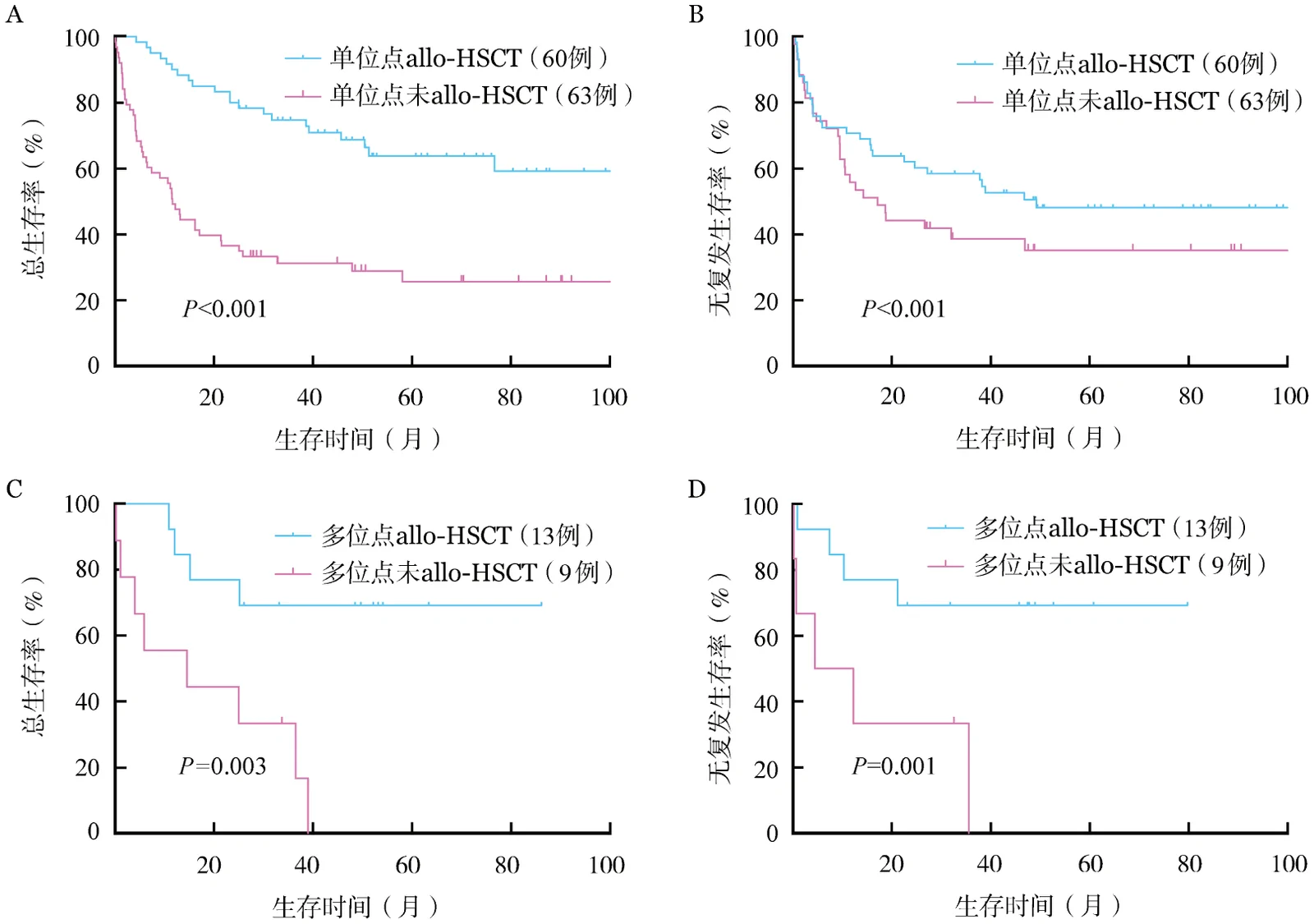
异基因造血干细胞移植（allo-HSCT）对伴RUNX1单位点与多位点基因突变急性髓系白血病患者生存的影响 **A** 单位点突变组总生存曲线；**B** 单位点突变组无复发生存曲线；**C** 多位点突变组总生存曲线；**D** 多位点突变组无复发生存曲线

单因素Cox回归分析显示，allo-HSCT是两组OS的共同的保护因素（*HR*＝0.283，95％ *CI*: 0.168～0.478，*P*<0.001；*HR*＝0.193，95％ *CI*: 0.057～0.654，*P*＝0.008），亦是两组RFS的共同保护因素（*HR*＝0.461，95％ *CI*: 0.287～0.740，*P*＝0.001；*HR*＝0.169，95％ *CI*: 0.049～0.579，*P*＝0.005）。多因素分析校正年龄、染色体核型、共突变基因数、融合基因等混杂因素后，allo-HSCT仍是两组OS的独立保护因素（*HR*＝0.256，95％ *CI*: 0.137～0.479，*P*<0.001；*HR*＝0.164，95％ *CI*: 0.029～0.944，*P*＝0.043），亦是两组RFS的独立保护因素（*HR*＝0.528，95％ *CI*: 0.302～0.924，*P*＝0.025；*HR*＝0.142，95％ *CI*: 0.024～0.828，*P*＝0.030）。

7. RUNX1与共突变基因的交互作用：根据共突变基因数量（≥3个对<3个）分组，两组患者的中位OS时间差异无统计学意义（25.17个月对76.67个月，*P*＝0.131）。RUNX1与TP53共突变患者的中位OS时间仅为6.5个月，显著短于不伴TP53突变的患者（47.9个月，*P*＝0.010）；伴DNMT3A共突变的患者的中位OS时间与不伴DNMT3A突变者差异无统计学意义（51.3个月对21.4个月，*P*＝0.770）；伴ASXL1共突变患者的中位OS时间与不伴ASXL1突变者差异无统计学意义（45.67个月对39.20个月，*P*＝0.805）（**扫描本文首页二维码查阅附图1**）。

## 讨论

RUNX1作为人类造血的重要调控基因，又称AML1，是转录因子核心结合因子家族的一员，位于染色体21q22区，包含12个外显子，全长262 kb，其编码蛋白为异二聚体转录因子的α亚基，包含三个关键结构域：N端高度保守的Runt同源结构域（RHD）、C端反式激活结构域（TAD）及转录抑制结构域（RD），RHD识别并结合特定的DNA序列，促进RUNX1转录因子的核定位，同时负责与核心结合因子β异二聚化形成转录因子，调控造血细胞增殖分化相关的信号通路[7]。TAD介导转录激活，RD参与转录抑制，二者共同精细调控RUNX1的转录活性[8]。Kirtek等[9]发现RUNX1突变的VAF对患者生存期无显著影响，本研究中，RUNX1突变位点个数及VAF均未对患者的缓解率及远期预后造成显著影响。因此，仅从突变数量或丰度层面难以全面评估RUNX1突变的致病性。Ito等[10]指出，RHD的错义突变常通过显性负效应干扰野生型RUNX1的功能，TAD突变（尤其是无义突变）可导致单倍剂量不足。不同功能域突变可能对疾病预后产生差异化影响，但由于突变位点所在功能域信息的缺失，本研究未能进行分层分析。未来研究可进一步定位突变功能域，评估其对治疗反应及远期预后的影响。

RUNX1突变与AML患者较低的诱导缓解率及生存率密切相关[11]–[12]。Speidel等[13]发现RUNX1直接参与了造血细胞对细胞毒性药物的反应，野生型RUNX1在阿糖胞苷处理后上调表达，进而抑制细胞增殖、促进细胞凋亡并增强DNA损伤反应，而突变型RUNX1由于RHD结构域破坏，失去抗增殖和诱导凋亡的活性，对化疗药物产生耐药性，既往研究发现，RUNX1突变的AML患者接受阿糖胞苷联合蒽环类药物的传统化疗方案缓解率低，VEN-HMA可能消除RUNX1的不良影响[14]–[16]。Mill等[17]发现，蛋白质翻译抑制剂（高三尖杉酯碱）与VEN联用对RUNX1突变的AML细胞具有协同杀伤作用，有望成为一种新的治疗选择。

与国外许多研究一致，本研究中RUNX1突变常与FLT3、DNMT3A、ASXL1等基因突变共同出现，与NPM1相排斥[18]–[19]。既往机制研究表明，RUNX1突变细胞本身存在生存和植入劣势，需要额外的基因改变才能存活和持续存在[20]，进一步解释了本研究中多位点组共突变基因数量显著高于单位点组（*P*＝0.001），提示RUNX1突变可能通过招募更多协同突变来驱动疾病进展。我们进一步分析了RUNX1与TP53、ASXL1、DNMT3A共突变对患者预后的影响，结果显示，TP53突变与RUNX1突变患者的极差预后显著相关（*P*＝0.010），表明TP53是RUNX1突变患者不良预后的协同突变基因；ASXL1与RUNX1共突变未显示出预后价值（*P*＝0.805），与Nguyen等[21]的研究一致，该研究还发现DNMT3A与RUNX1共突变对患者的OS有不良影响，在本研究中，DNMT3A共突变患者的OS呈不良趋势，但差异无统计学意义（*P*＝0.770），可能与样本量或随访时间有关。此外，Nguyen等[21]还观察到，共突变基因数量≥3个的RUNX1突变患者的OS时间显著短于<3个者（*P*＝0.004），本研究按此分组亦观察到类似趋势，但差异无统计学意义（*P*＝0.131），可能需要进一步扩大样本量研究论证。上述结果提示，RUNX1突变AML患者的不良预后是与其他共突变基因协同作用的结果。

本研究中，allo-HSCT显著改善了单位点组和多位点组患者的OS及RFS，Chou等[22]发现，RUNX1突变在非移植患者中为独立不良预后因素，但在接受allo-HSCT的患者中表现为良好的独立预后价值。Waidhauser等[23]发现，在首次CR期移植的RUNX1突变组与野生型组的2年OS率差异无统计学意义。Ran等[24]在中国人群中也观察到相似结果。上述结果表明，allo-HSCT可显著改善RUNX1突变患者的预后，提示移植可能克服RUNX1突变的不良影响。

综上所述，RUNX1单位点与多位点突变的AML患者在临床特征、治疗反应及远期预后方面均无显著差异。allo-HSCT能显著改善两组患者的OS和RFS，是有效的治疗手段。

## Supplementary Material



## References

[1] Tang JL, Hou HA, Chen CY (2009). AML1/RUNX1 mutations in 470 adult patients with de novo acute myeloid leukemia: prognostic implication and interaction with other gene alterations[J]. Blood.

[2] Kihara R, Nagata Y, Kiyoi H (2014). Comprehensive analysis of genetic alterations and their prognostic impacts in adult acute myeloid leukemia patients[J]. Leukemia.

[3] Döhner H, Wei AH, Appelbaum FR (2022). Diagnosis and management of AML in adults: 2022 recommendations from an international expert panel on behalf of the ELN[J]. Blood.

[4] 中华医学会血液学分会白血病淋巴瘤学组 (2023). 成人急性髓系白血病(非急性早幼粒细胞白血病)中国诊疗指南(2023年版)[J]. 中华血液学杂志.

[5] 中国抗癌协会血液肿瘤专业委员会, 中华医学会血液学分会, 中华医学会病理学分会 (2018). 二代测序技术在血液肿瘤中的应用中国专家共识(2018年版)[J]. 中华血液学杂志.

[6] Huang XJ, Liu DH, Liu KY (2009). Treatment of acute leukemia with unmanipulated HLA-mismatched/haploidentical blood and bone marrow transplantation[J]. Biol Blood Marrow Transplant.

[7] Perry C, Eldor A, Soreq H (2002). Runx1/AML1 in leukemia: disrupted association with diverse protein partners[J]. Leuk Res.

[8] Sood R, Kamikubo Y, Liu P (2017). Role of RUNX1 in hematological malignancies[J]. Blood.

[9] Kirtek TJ, Chen W, Harris JC (2026). Acute leukemias of ambiguous lineage with RUNX1 mutations show similar prognosis compared to acute myeloid leukemia with RUNX1 mutations: a study from the bone marrow pathology group[J]. Am J Hematol.

[10] Ito Y (2004). Oncogenic potential of the RUNX gene family: ‘overview’[J]. Oncogene.

[11] Gaidzik VI, Teleanu V, Papaemmanuil E (2016). RUNX1 mutations in acute myeloid leukemia are associated with distinct clinico-pathologic and genetic features[J]. Leukemia.

[12] Yamato G, Shiba N, Yoshida K (2018). RUNX1 mutations in pediatric acute myeloid leukemia are associated with distinct genetic features and an inferior prognosis[J]. Blood.

[13] Speidel D, Wellbrock J, Abas M (2017). RUNX1 upregulation by cytotoxic drugs promotes apoptosis[J]. Cancer Res.

[14] Wan CL, Huang YH, Huang SM (2024). Investigations of the prognostic value of RUNX1 mutation in acute myeloid leukemia patients: Data from a real-world study[J]. Leuk Res.

[15] 董 雨轩, 戴 文洁, 张 婉秋 (2026). RUNX1突变急性髓系白血病患者临床特征分析[J]. 中华血液学杂志.

[16] Venugopal S, DiNardo CD, Loghavi S (2022). Differential prognostic impact of RUNX1 mutations according to frontline therapy in patients with acute myeloid leukemia[J]. Am J Hematol.

[17] Mill CP, Fiskus W, DiNardo CD (2022). Effective therapy for AML with RUNX1 mutation by cotreatment with inhibitors of protein translation and BCL2[J]. Blood.

[18] Mendler JH, Maharry K, Radmacher MD (2012). RUNX1 mutations are associated with poor outcome in younger and older patients with cytogenetically normal acute myeloid leukemia and with distinct gene and MicroRNA expression signatures[J]. J Clin Oncol.

[19] Schnittger S, Dicker F, Kern W (2011). RUNX1 mutations are frequent in de novo AML with noncomplex karyotype and confer an unfavorable prognosis[J]. Blood.

[20] Rae DT, Hocum JD, Bii V (2015). A novel retroviral mutagenesis screen identifies prognostic genes in RUNX1 mediated myeloid leukemogenesis[J]. Oncotarget.

[21] Nguyen L, Zhang X, Roberts E (2020). Comparison of mutational profiles and clinical outcomes in patients with acute myeloid leukemia with mutated RUNX1 versus acute myeloid leukemia with myelodysplasia-related changes with mutated RUNX1[J]. Leuk Lymphoma.

[22] Chou SC, Tang JL, Hou HA (2014). Prognostic implication of gene mutations on overall survival in the adult acute myeloid leukemia patients receiving or not receiving allogeneic hematopoietic stem cell transplantations[J]. Leuk Res.

[23] Waidhauser J, Labopin M, Esteve J (2021). Allogeneic stem cell transplantation for AML patients with RUNX1 mutation in first complete remission: a study on behalf of the acute leukemia working party of the EBMT[J]. Bone Marrow Transplant.

[24] Ran WJ, Xu LP, Zhang XH (2025). Clinical effects of RUNX1 mutations on the outcomes of patients with acute myeloid leukemia treated with allogeneic hematopoietic stem-cell transplantation[J]. Curr Oncol.

